# Megalencephaly-Capillary Malformation-Polymicrogyria Syndrome (MCAP): A Rare Dynamic Genetic Disorder

**DOI:** 10.7759/cureus.25123

**Published:** 2022-05-18

**Authors:** Kalyan Sarma, Manoj K Nayak, Biswamohan Mishra, Shailesh B Gaikwad

**Affiliations:** 1 Neuroradiology, All India Institute of Medical Sciences, New Delhi, New Delhi, IND; 2 Neurology, All India Institute of Medical Sciences, New Delhi, New Delhi, IND

**Keywords:** phosphatidylinositol 3-kinase (pi3k)-akt pathway, megalencephaly, brain vascular malformation, mcap syndrome, megalencephaly-capillary malformation-polymicrogyria syndrome

## Abstract

Megalencephaly-capillary malformation-polymicrogyria syndrome (MCAP) is an uncommon malformation syndrome, characterized by primary megalencephaly, capillary malformations of the midline face and body, or distal limb anomalies such as syndactyly and polymicrogyria. Herein, we report a young male child, who presented with complaints of increasing head size, delay in speech, and one episode of focal seizure with distinctive morphological and neuroradiological manifestations which led to the diagnosis of MCAP. We have also reviewed recently published literature and the various diagnostic criteria proposed by authors to achieve the early clinical diagnosis of these patients in the outpatient department.

## Introduction

Megalencephaly-capillary malformation-polymicrogyria syndrome (MCAP) is an uncommon genetic syndrome characterized by primary megalencephaly, cutaneous vascular malformations, polymicrogyria, and other anomalies [1]. This condition was first described in 1997 as macrocephaly-cutis marmorata telangiectasia congenita (M-CMTC) by Clayton-Smith et al. [2] and Moore et al. [3]. After diagnosis in 1997, around 300 cases have been reported in the literature [4]. In 2007, this condition was renamed macrocephaly-capillary malformation syndrome (MCM) by Toriello and Mulliken et al. [5] and Conway et al. [6] Finally, in 2012 Mirzaa et al. renamed MCM to megalencephaly-capillary malformation-polymicrogyria syndrome (MCAP) to reflect the abnormally large size of the brain and to highlight the importance of perisylvian polymicrogyria [1]. Current studies have found its genetic cause to be linked with the PI3K-AKT pathway [7]. Herein, we describe a case, that presented to us with both intracranial and cutaneous manifestations of MCAP. Clinicians should have a high degree of suspicion with regular follow-up as the entity has highly dynamic clinical manifestations.

## Case presentation

**Figure 1:**
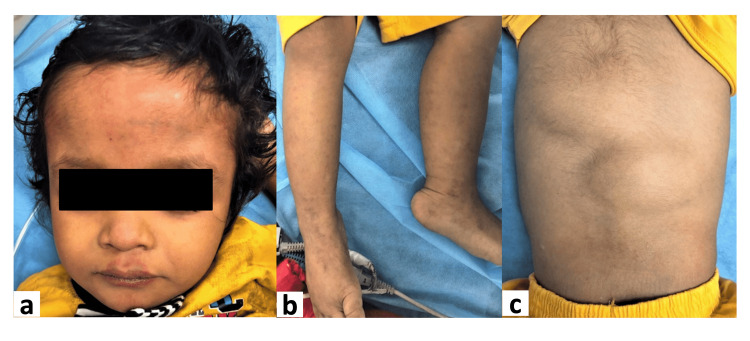
shows features seen on clinical examination of the patient Figure 1 shows the features found on general examination of the patient, macrocephaly with frontal bossing with cutaneous capillary malformations above the upper lip of face (1a), multiple cutaneous capillary malformations over bilateral lower limbs(1b), multiple thick doughy subcutaneous tissues over the back (1c).

A three-and-a-half-year-old male child presented with complaints of increasing head size [>2 Standard Deviation (2SD)], delay in speech, and one episode of focal seizure at three years of age. His perinatal history was uneventful. There was no family history of similar illnesses in the family. Examination revealed macrocephaly with frontal bossing (Figure 1a) with numerous cutaneous capillary malformations on the face and bilateral lower limbs (Figures 1a, 1b). Multiple thick doughy subcutaneous tissues were also present over the back (Figure 1c), which was confirmed as fibrofatty tissue on USG. No evidence of facial, body asymmetry, syndactyly, or polydactyly was found.

**Figure 2:**
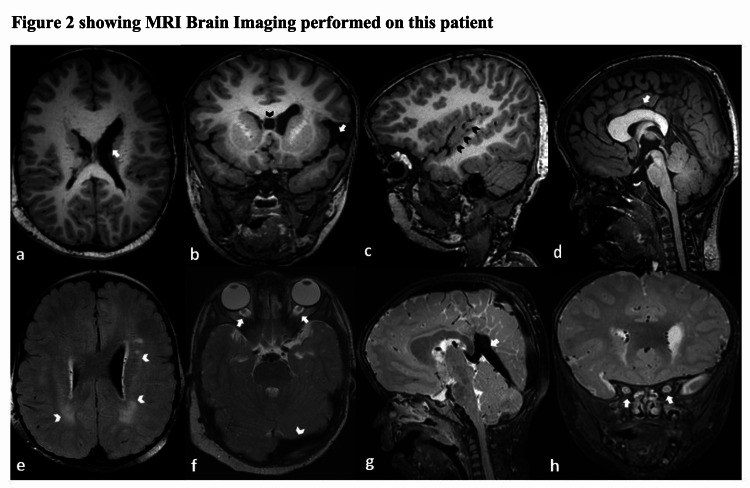
showing MRI Brain Imaging performed on this patient Figure 2 shows MRI Brain imaging performed on this patient. Axial T1W image reveals ventricular asymmetry with prominent left lateral ventricle (white arrow, Fig. 2a). Coronal T1W image reveals left-sided incomplete opercularization with widened left Sylvian fissure (white arrow, Fig. 2b), cavum septum pellucidum with prominent left lateral ventricle is also seen (black arrowhead Fig. 2b).T1W parasagittal image reveals perisylvian polymicrogyria (black arrowheads, Fig. 2c). T1W mid-sagittal image reveals abnormally thickened mega corpus callosum (white arrow, Fig. 2d). Axial FLAIR image demonstrates multiple foci of abnormally increased signals in bilateral deep and periventricular white matter (white arrowheads, Fig. 2e). Axial T2W image reveals prominent bilateral optic nerve sheaths (white arrows, Fig. 2f) and enlarged left transverse sinus flow void (white arrowhead, Fig. 2f). T2W mid-sagittal image reveals an enlarged straight sinus flow void (white arrow, Fig.2g). Coronal T2W image reveals prominent bilateral optic nerve sheaths (white arrows, Fig. 2h).

Magnetic resonance imaging (MRI) of the brain showed ventricular asymmetry with prominent left lateral ventricle (Figure 2a), left-sided incomplete opercularization with widened left sylvian fissure and cavum septum pellucidum (Figure 2b), bilateral perisylvian polymicrogyria (Figure 2c), abnormally thickened mega corpus callosum (Figure 2d), multiple foci of T2/FLAIR hyperintensities in bilateral deep and periventricular white matter (Figure 2e), prominent bilateral optic nerve sheaths (Figure 2f, 2h) and enlarged venous sinuses (Figure 2f, 2g). 

Based on the clinical and neuroimaging findings, the diagnosis of a megalencephaly-capillary malformation-polymicrogyria syndrome (MCAP) was made as per the proposed criteria by Mirzaa et al. with four core features (megalencephaly, capillary malformations in midline face and body, polymicrogyria and connective tissue dysplasia) and four supportive features (mega corpus callosum, the prominent venous system, frontal bossing and developmental delay) [1]. At present, parents are unwilling to perform a genetic work-up of the patient or his siblings fearing social stigmata, despite adequate assurances of maintaining the confidentiality of the genetic profiling results at our end. The child was managed with antiepileptic medication for control of seizures and provided with rehabilitation therapy for developmental delay, as well as is presently under follow-up. At the last follow-up at eight months, speech difficulty was persistent, however, he was seizure-free on a single antiepileptic. Due to his speech difficulty, his parents have been apprehensive about sending him to school, so they have been advised to enroll him in institutions meant for specially-abled children.

## Discussion

MCAP is a rare genetic syndrome characterized by a wide range of abnormalities like primary megalencephaly, cutaneous vascular malformations, prenatal overgrowth, connective tissue dysplasia, digital anomalies, body asymmetry with distinctive brain imaging features like polymicrogyria, asymmetry of the lateral ventricles, hydrocephalus, polymicrogyria, large cerebellum resulting in the crowded posterior fossa, cerebellar tonsillar herniation or ectopia, thick corpus callosum, and other features [1]. Riviere et al. [7] identified the de novo postzygotic or germline mutations in the AKT3, PIK3R2, PIK3CA genes to be associated with MCAP, and also suggested the substantial role of phosphatidylinositol 3-kinase (PI3K)-AKT pathway in the development of the brain, vasculature, and limbs. Familial MCAP is also reported, thereby advocating the germline mosaicism or autosomal recessive inheritance in parents [7].

A newer classification was proposed by Mirzaa et al, in 2012, which is commonly used for diagnosis [1]. Associated neuroimaging features reported in this disease include cerebral asymmetry, increased white matter signal, cavum septum pellucidum, ‘‘hydropic’’ appearing optic nerve sheaths, cortical dysgenesis/dysplasia, dilated perivascular spaces of the cortical veins, and venous sinus thrombosis [6]. The condition is associated with dynamic changes like - ventriculomegaly which may progress to hydrocephalus, cerebellar tonsillar ectopia, and mega corpus callosum because of which follows up with MRI is required [1]. In our case, there was the absence of supportive features like ventriculomegaly, and cerebellar tonsillar ectopia. Presently, no specific treatment for MCAP is available, but ARQ 092, an allosteric AKT inhibitor showed an antiproliferative effect on overgrowth syndromes including MCAP due to inhibition of the PI3K/AKT pathway [8]. Supportive management includes medical therapy for control of seizures and other symptoms, physiotherapy, speech, occupational and physical therapy to improve daily functioning. Surgical management includes shunt placement for hydrocephalus and posterior fossa decompression for symptomatic cerebellar tonsillar ectopia, especially with features of brainstem compression or syringomyelia [1].

Few similar MCAP cases were registered in the Johns Hopkins maintained Online Medillian Inheritance of Man (OMIM) with varied clinical and imaging manifestations. Initially, 13 unrelated cases were described in 1997 by Moore et al., with abnormalities in somatic growth, face, brain, vasculature, and connective tissue disorder and combined classified as megalencephaly-cutis marmorata telangiectasia congenita (MCMTC) [3]. Subsequently, nine additional patients were reported by Clayton-Smith et al. in 1997, [2] and Carcao et al. in 1998 where the latter supported the hypothesis that CNS and vascular dysgenesis leads to MCMTC [9]. Four additional cases were reported by Vogels et al. in 1998 [10], three cases by Yano and Watanabe et al. in 2001 [11], six patients by Lapunzina et al. in 2004, [12] seven patients by Giuliano et al. in 2004 [13], 10 patients by Garavelli et al. in 2005, [14] 17 patients by Conway et al. in 2007, [6] one patient by Canham and Holder in 2008, [15] three patients by Gripp et al. in 2009 [16]. As there were overlapping features between megalencephaly, polymicrogyria-polydactyly hydrocephalus syndrome, and capillary malformation, so Gripp et al., proposed the term megalencephaly-polydactyly-polymicrogyria-hydrocephalus capillary malformation (MPPH-CM) to this phenotypic spectrum [16]. Approximately 21 cases MPPH cases were also reported in the OMIM literature with the highest number of cases by Mirzaa et al. in 2014 [17] and there was significant phenotypic overlap and have a common genetic basis between these two by Nakamura et al. in 2014 [18].

Other simulating brain overgrowth syndrome includes megalencephaly-polydactyly-polymicrogyria-hydrocephalus (MPPH) syndrome, where megalencephaly is seen associated with distal limb anomalies like postaxial polydactyly and hydrocephalus [1]. Other diseases associated with megalencephaly and skin manifestations are congenital lipomatous overgrowth, vascular malformations, epidermal nevi (CLOVE) syndrome, and Bannayan-Riley-Ruvalcaba syndrome (BRRS) [19].

Even though imaging features like hydrocephalus and cerebellar tonsillar ectopia were absent at present in the present case, he is planned for regular follow-up with interval imaging at one year.

## Conclusions

Being a rare condition, MCAP requires careful clinical evaluation and neuroimaging for its diagnosis. Moreover, clinicians should be aware of its dynamic nature and so follow-up with MRI is required for cerebellar tonsillar ectopia and brainstem compression which may be life-threatening.
